# Application of a Hermite-based measure of non-Gaussianity to normality tests and independent component analysis

**DOI:** 10.3389/fninf.2023.1113988

**Published:** 2023-04-21

**Authors:** Parul Jain, Bruce W. Knight, Jonathan D. Victor

**Affiliations:** ^1^Weill Cornell Graduate School of Medical Sciences, New York, NY, United States; ^2^Feil Family Brain and Mind Research Institute, Weill Cornell Medical College, New York, NY, United States; ^3^Laboratory of Biophysics, The Rockefeller University, New York, NY, United States; ^4^Department of Neurology, New York Presbyterian Hospital, New York, NY, United States

**Keywords:** dimension reduction, EEG, Hermite functions, independent component analysis, non-Gaussianity, normality test, signal processing, source separation

## Abstract

In the analysis of neural data, measures of non-Gaussianity are generally applied in two ways: as tests of normality for validating model assumptions and as Independent Component Analysis (ICA) contrast functions for separating non-Gaussian signals. Consequently, there is a wide range of methods for both applications, but they all have trade-offs. We propose a new strategy that, in contrast to previous methods, directly approximates the shape of a distribution via Hermite functions. Applicability as a normality test was evaluated via its sensitivity to non-Gaussianity for three families of distributions that deviate from a Gaussian distribution in different ways (modes, tails, and asymmetry). Applicability as an ICA contrast function was evaluated through its ability to extract non-Gaussian signals in simple multi-dimensional distributions, and to remove artifacts from simulated electroencephalographic datasets. The measure has advantages as a normality test and, for ICA, for heavy-tailed and asymmetric distributions with small sample sizes. For other distributions and large datasets, it performs comparably to existing methods. Compared to standard normality tests, the new method performs better for certain types of distributions. Compared to contrast functions of a standard ICA package, the new method has advantages but its utility for ICA is more limited. This highlights that even though both applications—normality tests and ICA—require a measure of deviation from normality, strategies that are advantageous in one application may not be advantageous in the other. Here, the new method has broad merits as a normality test but only limited advantages for ICA.

## 1. Introduction

Tests for normality play many important roles in neural data analysis. Two of these are complementary. One such role is to determine whether a distribution is sufficiently close to normal (Wilcox and Rousselet, 2018; Kwak and Park, 2019) to justify an assumption of normality. A contrasting role is to identify distributions with the greatest deviation from normality (McKeown and Sejnowski, 1998; Vigário et al., 2000; James and Hesse, 2004; Onton et al., 2006). This is the key step in independent component analysis (ICA), and is motivated by the heuristic that unmixed sources have the lowest entropy, and are therefore highly non-Gaussian (Comon, 1994; Bell and Sejnowski, 1995). Perhaps as a consequence of these disparate goals, there are many tests of normality (Shapiro et al., 1968; Yazici and Yolacan, 2007) and related ICA contrast functions (Grouiller et al., 2007; Rejer and Górski, 2013), but each of these has trade-offs. Here we introduce a new method, and compare its utility to existing approaches for a range of simulated datasets.

The advantages and disadvantages of the various kinds of normality tests and ICA contrast functions can be understood in terms of the strategies that they use. For example, moment-based normality tests can be overly sensitive to outliers (Shapiro et al., 1968). Tests based on cumulative distribution functions and frequency statistics give appropriate weight to tails (Shapiro et al., 1968; Mendes and Pala, 2003), but may overlook the shape near the center. ICA contrast functions that utilize entropy-based measures face the difficulty of estimating entropy from limited data (Paninski, 2003).

Our strategy diverges from these methods: it is based on approximating the distribution of data by using a set of orthonormal basis functions, the Hermite functions (Szegö, 1939). In principle, since the coefficients of a Hermite expansion can be estimated via simple linear estimators that are insensitive to outliers, this approach provides advantages for some kinds of data distributions.

Here, we conduct a thorough investigation to assess whether a Hermite functions-based measure of non-Gaussianity is useful as a normality test and as an ICA contrast function. We note that similar approaches using Hermite functions have been previously proposed for moment-based normality tests (Almuzara et al., 2019; Amengual et al., 2022) and distribution shape-sensitive ICA (Puuronen and Hyvärinen, 2011), but a comparison to other normality tests or applicability as an ICA contrast function had not been undertaken. We test the proposed method by using datasets constructed to have simple distributions as well as realistic EEG simulations. We then benchmark the performance of our method against common normality tests and ICA contrast functions included in a popular ICA package, FastICA (Hyvärinen, 1999). Our method has advantages as a normality test for heavy-tailed and asymmetric distributions. However, as an ICA contrast function, these advantages are only realized for a niche of datasets: small datasets with components that have heavy tailed or asymmetric distributions.

## 2. Methods

We first introduce Hermite functions and the properties that underlie our proposed measure of non-Gaussianity. Next, we formulate the method which serves as a normality test and a contrast function for ICA. Finally, we detail the datasets and procedures we use for assessing the performance of the proposed method.

### 2.1. Background on Hermite functions

Our approach to assess the non-Gaussianity of a probability distribution *p*(*x*) centers on its expansion in terms of an orthonormal family, the Hermite functions *H*_*n*_(*x*) (Szegö, 1939). In the Hermite expansion of *p*(*x*), the first term, *H*_0_(*x*), is a Gaussian. This property allows the Hermite expansion to separate the Gaussian and the non-Gaussian parts of *p*(*x*).

Each Hermite function (*H*_*n*_(*x*)) is given by the Hermite polynomial of the same order (*h*_*n*_(*x*)), multiplied by a Gaussian envelope $e^{-\frac{x^{2}}{2}}$. In the standard convention used here, the Hermite polynomials *h*_*n*_(*x*) are orthogonal with respect to the Gaussian weight *e*^−*x*2^, and are given by


(1) $$\begin{array}{l} h_{n}\left(x\right)={\left(-1\right)}^{n}e^{x^{2}}\frac{d^{n}}{dx^{n}}e^{-x^{2}}. \end{array}$$

The Hermite function of order *n* (*H*_*n*_(*x*)) thus corresponds to the product of the Hermite polynomial of the same order (*h*_*n*_(*x*)) and a Gaussian with mean 0 and variance 1.


(2) $$\begin{array}{l} H_{n}\left(x\right)=\frac{1}{\sqrt{2^{n}n!\sqrt{\pi }}}e^{-\frac{x^{2}}{2}}h_{n}\left(x\right). \end{array}$$

With this definition, the orthonormality of Hermite functions is expressed by


(3) $$\int_{-\infty }^{\infty }H_{m}(x)\;H_{n}(x)\;dx=\delta_{mn}.$$

This property allows us to express probability distribution functions *p*(*x*) in terms of Hermite functions:


(4) $$\begin{array}{l} p\left(x\right)=\overset{\infty }{\underset{i=0}{\sum }}a_{i}H_{i}\left(x\right). \end{array}$$

If *p*(*x*) is known, the coefficients *a*_*i*_ can be found in the following manner:


(5) $$a_{i}=\int_{-\infty }^{\infty }H_{i}(x)\;p(x)\;dx.$$

This evaluation of the coefficients *a*_*i*_ follows from the orthonormality property of Hermite functions:


(6) $$\int_{-\infty }^{\infty }H_{i}(x)\;p(x)\;dx\;=\;\int_{-\infty }^{\infty }H_{i}(x)\sum_{j=0}^{\infty }a_{j}H_{j}(x)\;=\;\int_{-\infty }^{\infty }a_{i}H_{i}(x)H_{i}(x)\;=a_{i}.$$

### 2.2. Hermite function-based measure of non-Gaussianity

In the Hermite expansion of *p*(*x*) (Equation 4), *H*_0_ is Gaussian (Equation 2), and thus its coefficient *a*_0_ can be considered to represent the Gaussian part of *p*(*x*). We leverage this to estimate non-Gaussianity of *p*(*x*) via the coefficients of the higher-order Hermite functions (*H*_*i*_(*x*) where *i*≠0) that enter into its Hermite expansion (Equation 4). Specifically, we propose to measure non-Gaussianity as the relative power in the coefficients corresponding to all but the zeroth order Hermite function:


(7) $$\begin{array}{l} J=1-\frac{a_{0}^{2}}{\overset{\infty }{\underset{i=0}{\sum }}a_{i}^{2}}. \end{array}$$

The minimum possible value of *J* is 0, which is only achieved for a Gaussian distribution with mean 0 and variance 1, i.e., when *a*_0_ is non-zero and all other *a*_*i*_ are 0. Positive values of *J* indicate a deviation from this distribution.

However, this measure cannot be used directly for three reasons. The first reason is that our data might not have 0 mean and 1 variance. We address this by standardizing our data before we estimate *J*. The second reason is that the estimation of *a*_*i*_ uses *p*(*x*) (Equation 5), which is unknown. Instead, we estimate the $a_{i}^{est}$ from samples *x*_*j*_ drawn from *p*(*x*):


(8) $$\begin{array}{l} a_{i}^{est}=\overset{N}{\underset{j=1}{\sum }}\frac{H_{i}\left(x_{j}\right)}{N}. \end{array}$$

The third reason is that the infinite sum in the denominator of equation 7 is divergent for distributions which are not square integrable (such as a discrete distribution based on a finite number of samples). Thus, we modify the estimator in Equation (7) by truncating the denominator to a finite number of terms *n*:


(9) $$\begin{array}{l} J_{n}=1-\frac{{\left(a_{0}^{est}\right)}^{2}}{\overset{n}{\underset{i=0}{\sum }}{\left(a_{i}^{est}\right)}^{2}}. \end{array}$$

By limiting the number of Hermite coefficients, we trade off resolution of the shape of the distribution for an estimator that is robust and avoids overfitting—a kind of regularization. As we show in the Section 3, the choice *n* = 15 works well in many practical situations, and we use that value unless otherwise stated.

Here, we examined two applications of *J*_15_. First, as a normality test, we compare its ability to detect deviations from a normal distribution with that of standard normality tests, and to the measures of normality typically used as ICA contrast functions. Second, since performance as a test of normality need not necessarily translate into utility as a contrast function in ICA, we directly compared an implementation of ICA that uses *J*_15_ as a contrast function with standard ICA (Hyvärinen, 1999), for simple low-dimensional datasets with known statistics and in realistic simulated EEG datasets.

### 2.3. Evaluation of *J*_15_ as a normality test

To determine the sensitivity of *J*_15_ to typical ways in which distributions may deviate from the normal distribution, we compared its ability to detect non-Gaussianity for three families of distributions: a family of distributions that differed in the extent of bimodality, a family of unimodal symmetric distributions that ranged from light- to heavy-tailed, and a family of unimodal distributions that varied in the degree of asymmetry.

#### 2.3.1. Bimodal distributions

To examine sensitivity to bimodality, we used a family of distributions consisting of a mixture of Gaussians (Equation 10) with a parameter α that controls the mixing weights.


(10) $$\begin{array}{l} p\left(x\right)=\alpha N\left(x;\mu_{1},\sigma_{1}\right)+\left(1-\alpha \right)N\left(x;\mu_{2},\sigma_{2}\right) \end{array}$$

where *N*(*x*; μ_*i*_, σ_*i*_) denotes normally distributed random variable *x* with mean μ_*i*_ and standard deviation σ_*i*_. The distribution is Gaussian for α = 0, or 1 and bimodal for intermediate values.

#### 2.3.2. Light- and heavy-tailed distributions

To examine the gamut from light- to heavy-tailed distributions, we used the family of symmetric generalized normal distributions (Equation 11).


(11) $$p(x)=C(\beta )\;e^{-\frac{|x{|}^{\beta }}{2}};$$

where *C*(β) is a normalizing constant. This distribution is Gaussian for β = 2, light-tailed (platykurtic) for β>2, and heavy-tailed (leptokurtic) for β < 2.

#### 2.3.3. Unimodal asymmetric distributions

To examine sensitivity to asymmetry, we used the family of generalized extreme value distributions (Equation 12).


(12) $$p(x)=e^{-{\left(1+\kappa x\right)}^{-\frac{1}{\kappa }}}{\left(1+\kappa x\right)}^{-1-\frac{1}{\kappa }}.$$

This distribution has a heavier left tail for negative values of κ, a heavier right tail for positive values of κ, and a transition near −0.3.

#### 2.3.4. Procedure

For each of the above families, we estimated non-Gaussianity via *J*_15_ for a range of distribution shapes (generated by varying the family parameter α, β, or κ), and compared these values with measures provided by five standard tests of normality and three standard ICA contrast functions. These comparisons were repeated for 1,000 runs of simulated data, and different sizes of datasets (10^3^, 10^4^, and 10^5^).

While there are many normality tests available today, we focused on five of the most common tests that use a range of strategies. Among the standard normality tests used, two tests were based on cumulative distribution functions (Kolmogorov-Smirnov test Kolmogorov, 1933 and Anderson-Darling test Anderson and Darling, 1952), two were moment-based (Jarque-Bera test Jarque and Bera, 1987 and D'Agostino's k-squared test D'Agostino et al., 1990), and one used frequency statistics (Shapiro-Wilk test Shapiro and Wilk, 1965).

The ICA contrast functions compared here are part of a standard ICA package, FastICA (Hyvärinen, 1999). These comparisons were chosen for two reasons. First, we are targeting analysis of EEG datasets; FastICA is commonly used for this purpose (Vigário, 1997; Rejer and Górski, 2013). The second reason relates to the strategy taken by the new method: it finds components one by one, as does FastICA (via the “deflate” argument for approach). We did not compare our approach to methods (Amari et al., 1995; Belouchrani et al., 1997) that find all components simultaneously, as the proposed contrast function, in its present form, cannot do this. Details of the ICA contrast functions used are discussed in Section 2.4.

### 2.4. Evaluation of *J*_15_ as a contrast function in ICA

To assess the applicability of *J*_15_ to ICA, we evaluate the accuracy and precision with which it could identify the maximally non-Gaussian 1D projections of 2- and 5-dimensional datasets with simple density functions, and in high-dimensional datasets drawn from a standard biologically-realistic simulation of EEG data. 1D projections of the 2D datasets were further used to find the optimal number of Hermite functions required in Equation (9) to assess non-Gaussianity. In each case, the true independent components were known, so we could compare accuracy and precision with standard ICA measures used in FastICA (Hyvärinen, 1999), and the dependence of these measures on the size of the dataset.

The standard ICA contrast functions used were: *FastICA*-*I*, which is kurtosis-based (invoked with the argument pow3), and *FastICA*-*II* and *FastICA*-*III*, which are two approximations of differential entropy (invoked with the arguments tanh and gaus). They are defined by


(13) $$\begin{array}{l} FastICA\text{-}I\left(X\right)=\frac{1}{4}E\left[X^{4}\right], \end{array}$$

(14) $$\begin{array}{l} FastICA\text{-}II\left(X\right)=E\left[log\left(cosh\left(X\right)\right)\right], \end{array}$$

and


(15) $$FastICA\text{-}III(X)=E[-e^{-\frac{X^{2}}{2}}].$$

#### 2.4.1. 2D and 5D datasets

In the two-and five-dimensional datasets, the component variables were independently drawn from a Gaussian distribution or a member of one of the above families (Section 2.3), with the following parameters: bimodal symmetric (α = 0.5;*N*(*x*; −2, 1) and *N*(*x*; 2, 1)), bimodal asymmetric (α = 0.7;*N*(−2, 1) and *N*(*x*; 2, 0.4)), heavy-tailed (β = 1), light-tailed (β = 10), and unimodal asymmetric (κ = 0).

Two-dimensional datasets were generated by sampling a product distribution that was Gaussian along one axis and non-Gaussian along the orthogonal axis. For each dataset sampled from the distribution, an exhaustive search (adaptive step size from 1° down to 0.1°) was done to estimate the direction of maximal non-Gaussian projection of data, i.e., the direction that yielded the maximum value of *J*_15_ or one of the standard contrast functions. This direction is referred to as the estimated direction of unmixing and its convergence to the true non-Gaussian axis of the underlying distribution measures accuracy. Precision is measured from the estimated direction of unmixing, i.e., the direction at which non-Gaussianity is maximal. Specifically, precision is the angular deviation from this direction at which estimates of non-Gaussianity first reliably deviate from maximal non-Gaussianity.

1D projections of these 2D datasets were also used to find an appropriate number of Hermite functions for estimating non-Gaussianity in Equation (9). We used these datasets for two reasons: (i) they allow us to test for different aspects of non-Gaussianity, and ii) they allow for an exhaustive search. That is, we can determine the precision with which they identify the maximally non-Gaussian direction, which is critical to ICA. For this analysis, we varied the highest order of Hermite functions *n* in Equation (9) from 2 to 50, and, for each value of *n*, we considered variants in which the denominator included (a) all orders up to *n*, (b) even orders up to *n*, and (c) odd orders up to *n* (but also including *n* = 0). Dataset sizes were 10^2^, 10^3^, 10^4^, and 10^5^ samples. An exhaustive search similar to 2D datasets was done to find the estimated direction of unmixing, but with a smaller range of angles (−10° to 10°, with step-size 0.1° for −2° to 2°, and 0.5° otherwise). Error is defined as the average angle between the estimated direction of unmixing and the true direction of unmixing. Confidence limits for error were determined from 1,000 bootstraps.

The 5D datasets were generated by sampling a product distribution that was non-Gaussian along one axis and Gaussian along the orthogonal axes. Since an exhaustive search was impractical, we modified the standard FastICA algorithm (Hyvärinen, 1999) to use a Hermite-based contrast function. This required modification of the search algorithm of FastICA to gradient descent with a time-based learning schedule, since the standard search algorithm requires the contrast function to be linear in the distribution, but *J*_15_ is not. To increase the likelihood that the search converged to an extremum for all contrast functions, we used 25 random starts and selected the best result for analysis. For a pilot subset of distributions, using 100 random starts did not result in further improvement. These differences in search algorithm increased the computational cost of *J*_15_ by more than a factor of 20; for some of the larger datasets analyzed here, this factor was more than 1,000. We took a final step to ensure that any apparent difference in performance of the contrast functions was due to the contrast function itself and not due to differences in the search algorithm: we chose, for each contrast function, the direction at which it achieved its extreme value, including consideration of the directions identified by extremizing the other contrast functions.

Error and precision were assessed with measures that were analogous to those used in the 2D datasets for the estimation of an optimum set of Hermite functions. Error was quantified by the angle between the estimated and true directions of unmixing. Precision was quantified by the angular variance, a generalization of the circular variance to higher-dimensional distributions. In general, for a set of *N* unit vectors *U*, the angular variance *av*(*U*) is defined as:


(16) $$av(U)=1-R;\;where\;R^{2}=|\frac{1}{N}\sum_{j=1}^{N}\vec{u_{j}}{|}^{2}.$$

Since an estimated direction of unmixing ${\vec{u}}_{est}$ and its negative cannot be distinguished by ICA, we replaced each estimated direction of unmixing by ${\vec{u}}_{est}={\vec{u}}_{est}sgn\left({\vec{u}}_{est}.{\vec{u}}_{true}\right)$ before applying Equation (16).

#### 2.4.2. Simulated EEG datasets

To test the approach on datasets that have a level of complexity comparable to biological data (e.g., EEG and the artifacts that corrupt EEG signals) yet also have a known ground truth, we used the simBCI package (Lindgren et al., 2018) to generate 249-electrode EEG data based on a realistic head model. The simulation included ocular artifacts referred to in the paper as eye blinks generated by each eye. We tested the ability of ICA to identify the directions corresponding to these artifacts. Simulations were 5 min long and sampled at 250 Hz. To assess the effect of the quantity of data, we applied ICA to 6-s, 15-s, 30-s, 1-min, 2.5-min, and 5-min segments, and analyzed 30 examples of each run length. As is standard, principal compoenent analysis was applied prior to ICA for an initial dimensionality reduction; we retained 50 principal components accounting for over 99% of the variance observed. Finally, each ICA run produced 20 components from which the two components corresponding to ocular artifacts were determined by visual inspection of the waveforms. Accuracy and precision were measured as described above for the 5D datasets, separately for the signals associated with each eye.

## 3. Results

Below, we explore the use of Hermite-based contrast function as a normality test and a viable option for ICA. As detailed in Section 2, the approach capitalizes on the fact that Hermite functions form an orthonormal basis set, in which one basis vector, the zeroth-order Hermite function, is a Gaussian. Thus, a probability distribution can be expressed as a linear sum of Hermite functions, with the zeroth order term representing the Gaussian part of the distribution. This allows us to measure non-Gaussianity by comparing the size of the zeroth-order coefficient to the size of higher-order coefficients, as formalized by Equation (9).

We assess the performance of this measure in two kinds of applications: as a normality test of one-dimensional distributions, and as a contrast function in ICA for multi-dimensional datasets. The tests of applicability to ICA use simple two- and five-dimensional datasets with known distributions and high-dimensional simulated EEG datasets with ocular artifacts.

### 3.1. Comparison of measure of non-Gaussianity to normality tests and contrast functions

We evaluate the potential use of *J*_15_ as a measure of non-Gaussianity by measuring its sensitivity to typical deviations from the normal distribution. To this end, we used three families of one-dimensional datasets deviating from normality in different aspects of the distribution: modes, heaviness of tails, and symmetry. For each family, a range of distributions with varying shape were tested. We expect that within a family, a good measure of non-Gaussianity will grow as the distribution shape becomes more non-Gaussian. To determine the advantage of the proposed method over the existing ones, we compared its performance against standard normality tests and contrast functions included in a popular ICA package (Hyvärinen, 1999).

**Figure 1:**
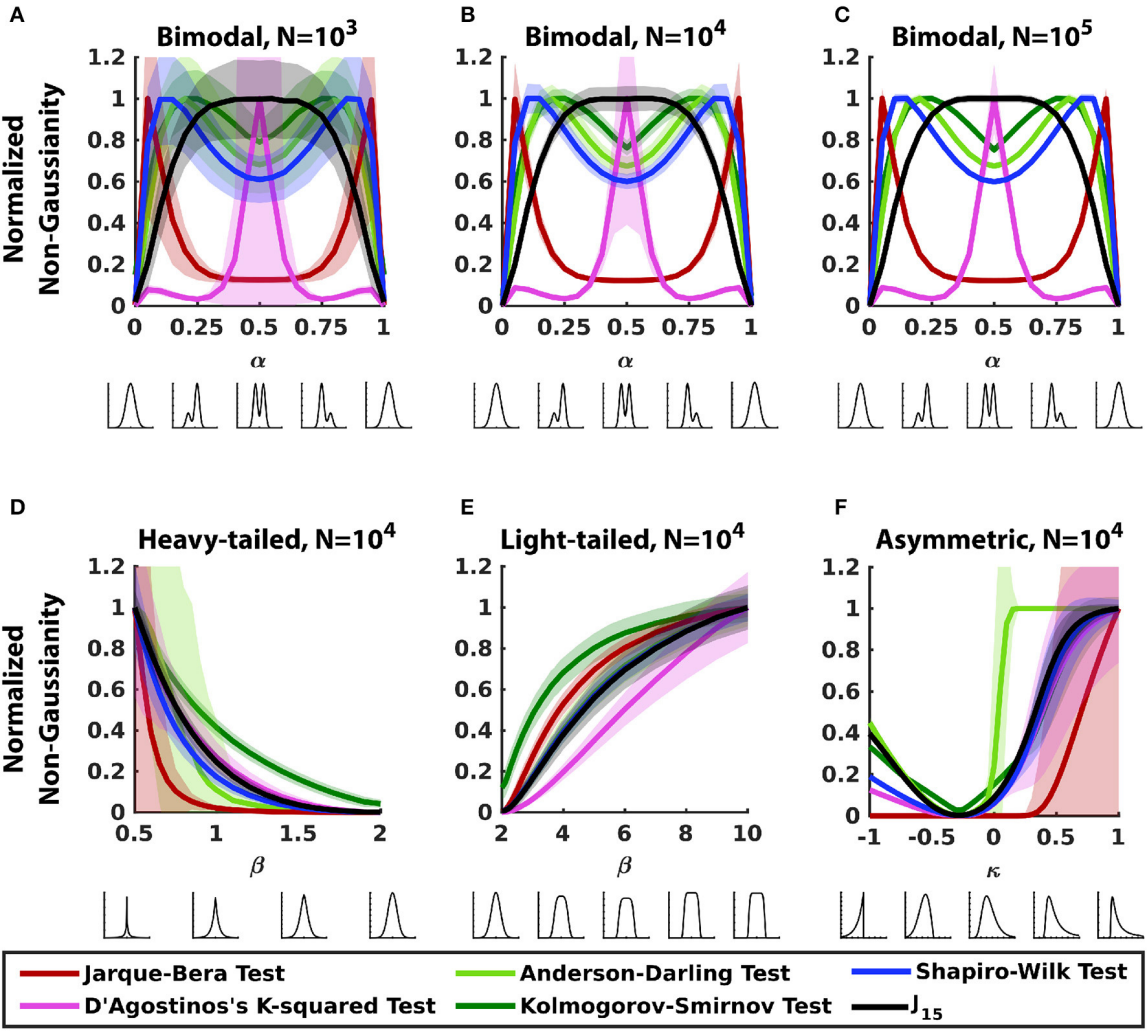
Comparing sensitivity of *J*_15_ and standard normality tests for 1D datasets. Each panel analyzes a parametric family of distributions, shown below each abscissa (see Section 2.3 for details). **(A–C)** Bimodal distributions (Section 2.3.1) with a range of sample sizes. **(D)** Heavy-tailed distributions (Section 2.3.2). **(E)** Light-tailed distributions (Section 2.3.2). **(F)** Asymmetric distributions (Section 2.3.3). Within each panel, measures were normalized so that the maximum value of the mean is 1. Error bars correspond to ± 2*SD*.

Figure 1 compares the sensitivity of *J*_15_ for non-Gaussianity to standard normality tests for the three families described in Section 2.3.

For bimodal distributions (Figures 1A–C), the Hermite-function measure, as expected, identifies the bimodal distribution as maximally non-Gaussian, and indicates that non-Gaussianity decreases as bimodality becomes less prominent. However, the standard tests of normality deviate from this behavior, and the way that they deviate can be understood in terms of the approach these tests employ. Specifically, the moment-based tests are heavily influenced by the tails and symmetry of the distributions: the D'Agostino's k-squared test, which is dominated by kurtosis performs poorly for distributions that are skewed (asymmetric bimodal), while the Jarque-Bera test, in which skewness has a heavier weighting, performs poorly for symmetric bimodal distributions. Note that though skewness and kurtosis both peak near the ends (α = 0, 1), D'Agostino k-squared test peaks for bimodal distribution (α = 0.5) and has only minor peaks near the ends. Tests based on cumulative distribution functions (Kolmogorov-Smirnov test and Anderson-Darling test) measure non-Gaussianity via the accumulated difference between the distributions of the data and a Gaussian: asymmetric distributions accumulate the difference faster resulting in greater sensitivity to non-Gaussianity for asymmetric distributions than for bimodal distributions. As tests based on frequency statistics (Shapiro-Wilk test) use principles intuitively similar to tests based on cumulative distribution functions, they behave similarly. Note also that these differences in behavior persist over a wide range of sample sizes (10^3^ to 10^5^ in Figures 1A–C). We also examined the performance of the Lilliefors test and the Cramer-von Mises criterion; these are related to the Kolmogorov-Smirnov test and performed similarly to it (not shown) for these distributions and the others described below.

For heavy-tailed and light-tailed distributions (Figures 1D, E), all measures track non-Gaussianity and increase as the distribution deviates further from a normal distribution. For these symmetric distributions, moment-based tests rely solely on kurtosis since their skewness is zero. Despite this, the two moment-based tests have opposite performance for the two sets of distributions: the Jarque-Bera test performs poorly for light-tailed distributions and D'Agostino's k-squared test performs poorly for heavy-tailed distributions. This can be attributed to the way kurtosis is used by the two tests. In the Jarque-Bera test, kurtosis is squared, therefore it grows rapidly and is more sensitive to heavy-tailed distributions. D'Agostino k-squared test uses a function involving the cube root of kurtosis, which decreases slower than kurtosis for heavy tailed distributions but is approximately a linear function of family parameter β for light-tailed distributions. By virtue of the symmetry and unimodality of these distributions, tests based on cumulative-distribution (Kolmogorov-Smirnov test and Anderson-Darling test) and frequency statistics (Shapiro-Wilk test) perform as well as or better than *J*_15_. These trends persisted when sample size was varied (data not shown).

For asymmetric distributions (Figure 1F), all measures track non-Gaussianity but the standard normality tests have low precision, as indicated by wide error bars. Both moments-based tests perform poorly for distributions with heavier left tails, but for distributions with heavier right tail, D'Agostino's k-squared test performs better than Jarque-Bera test despite the latter giving more weight to skewness. This is because for this family of distributions, kurtosis rises faster with κ than skewness. Tests based on cumulative distribution function and frequency statistics quickly accumulate the difference between the cumulative data distribution and a Gaussian distribution and perform with good accuracy but less precision.

**Figure 2:**
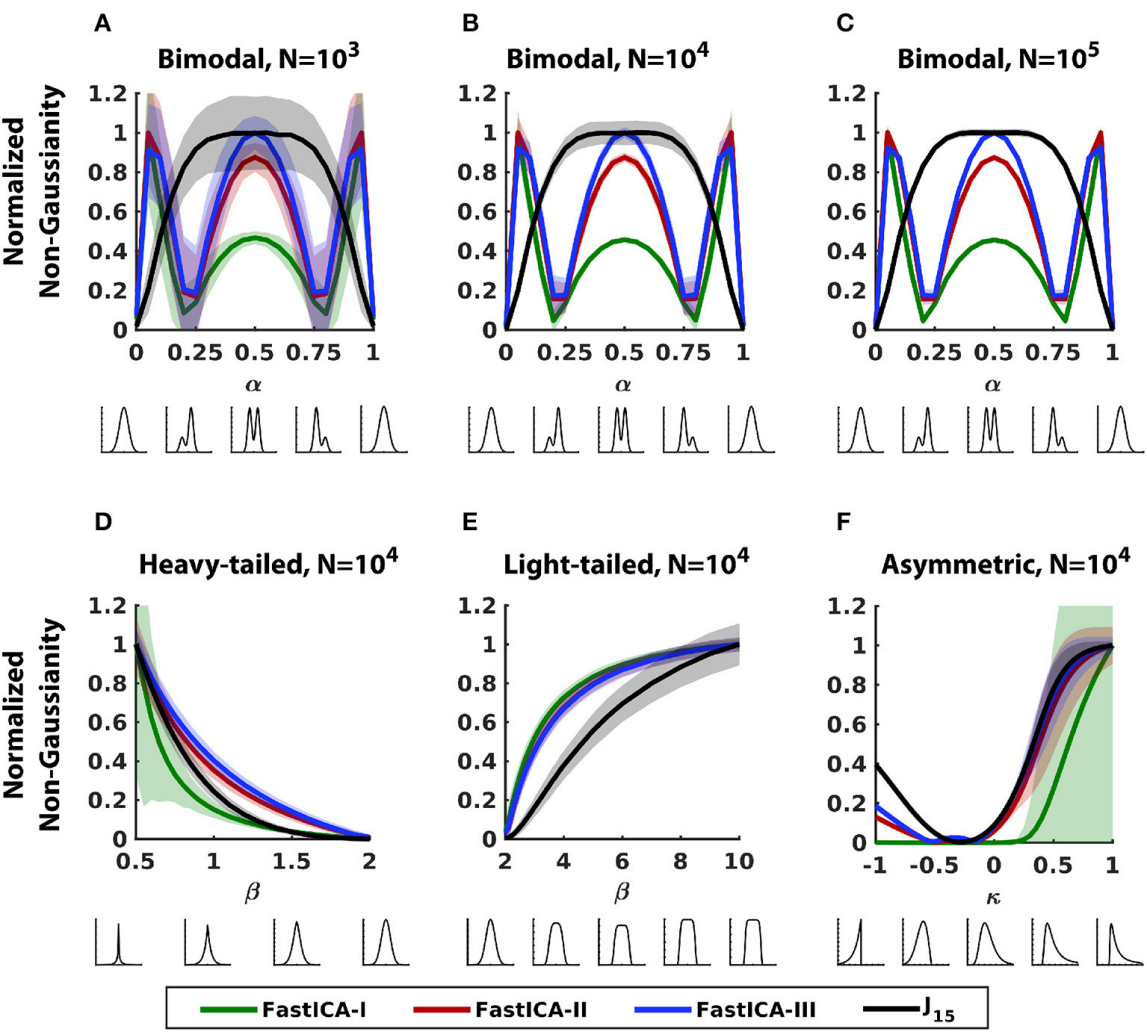
Comparison of the sensitivity of *J*_15_ to that of FastICA contrast functions for 1D datasets. **(A–C)** Bimodal distributions (Section 2.3.1) with a range of sample sizes. **(D)** Heavy-tailed distributions (Section 2.3.2). **(E)** Light-tailed distributions (Section 2.3.2). **(F)** Asymmetric distributions (Section 2.3.3). Other conventions as in Figure 1.

Figure 2 extends this analysis to the three contrast functions typically used in FastICA (Hyvärinen, 1999): the default function, based on kurtosis (FastICA-I), and two options based on approximations of entropy (FastICA-II and III). Results show that the performance of all three contrast functions has many features in common with that of the moment-based methods (Figure 1 in red and pink). For example, in bimodal distributions (Figures 2A–C), the measure of non-Gaussianity peaks either for bimodal symmetric distribution in the center (α = 0.5) like Jarque-Bera test or for asymmetric bimodal distributions near the ends (α = 0, 1) like the D'Agostino's k-squared test. Consequently, they are relatively insensitive to deviations from non-Gaussianity in asymmetric bimodal distributions (α~0.25 or ~0.75). For heavy-tailed distributions (Figure 2D), FastICA-I behaves similarly to Jarque-Bera test (low accuracy and precision) but FastICA-II and III perform similarly to D'Agostino's k-squared test (good accuracy and precision). For light-tailed distributions (Figure 2E), all three FastICA methods perform similarly to D'Agostino's k-squared test and perform better than *J*_15_ with respect to precision and accuracy. For asymmetric distributions (Figure 2F), FastICA-I performs similarly to Jarque-Bera test (low accuracy and precision) and FastICA-II and III perform similarly to D'Agostino's k-squared test (good accuracy but low precision).

### 3.2. Utility as a contrast function in independent component analysis

To assess the utility of a Hermite-based measure of non-Gaussianity (*J*_15_) as a contrast function for ICA, we compared its performance against the ICA contrast functions used in Figure 2 for multi-dimensional datasets. For simple 2D datasets, we compare accuracy and precision in an exhaustive search and further use this framework to choose the set of Hermite functions for estimation of non-Gaussianity using Equation (9). For simple 5D datasets and simulated EEG datasets, we compare accuracy using ICA implementation of the contrast functions in FastICA (Hyvärinen, 1999).

#### 3.2.1. 2D datasets

We generated 2D datasets by mixing a Gaussian and a non-Gaussian component (Section 2.4). The non-Gaussian components were drawn from the three families of distributions analyzed in Figures 1, 2 which represent different aspects of non-Gaussianity (bimodality, heaviness of tails, and asymmetry). Details of the distributions are provided in Section 2.4.

**Figure 3:**
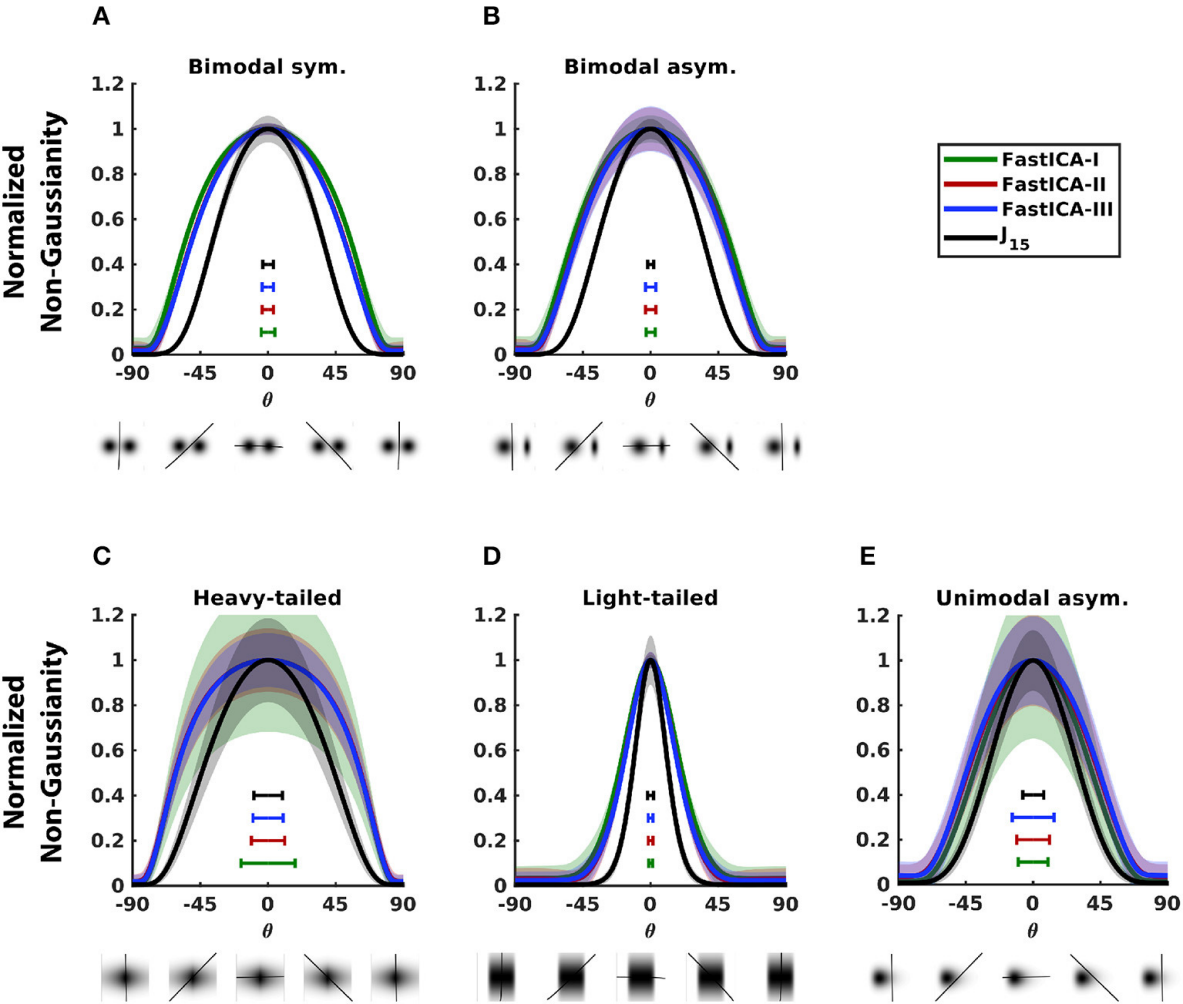
Comparison of the performance of *J*_15_ to that of contrast functions of FastICA for 2D distributions. Each panel analyzes a distribution consisting of a mixture of a Gaussian and a non-Gaussian distribution chosen from one of the families shown in Figures 1, 2, and is illustrated below each abscissa (see Section 2.4 for details). Non-Gaussianity is assessed for projections onto a range of angles, also illustrated below the abscissae. Results shown for 10^4^ samples. **(A)** Bimodal symmetric distribution. **(B)** Bimodal asymmetric distribution. **(C)** Heavy-tailed distribution. **(D)** Light-tailed distribution. **(E)** Unimodal asymmetric distribution. Normalization and error bars reported as in Figure 1. For each measure, accuracy is indicated by peak location and precision is inversely related to peak width, as indicated by the horizontal bars within each panel. For further details, see Section 2.3.

For 2D distributions, all methods were accurate (Figure 3) but *J*_15_ had the best precision for most distributions. The precision for FastICA contrast functions followed trends observed for 1D datasets in Figure 2. For bimodal distributions (Figures 3A, B), FastICA contrast functions have wider peaks than *J*_15_, corresponding to the finding in 1D distributions (Figures 2A–C) that these methods have reduced sensitivity for structure near the center. For heavy-tailed distributions, FastICA methods have low precision (width of peak) and for light-tailed distributions, they have high precision. This behavior of FastICA methods is due to their over-sensitivity to weight in tails. For unimodal asymmetric distributions, as was seen for 1D datasets, *J*_15_ has better precision than FastICA contrast functions (Figure 3E). As expected, precision increased with the number of samples but the differences between methods persisted (data not shown).

#### 3.2.2. Effect of number of Hermite functions

**Figure 4:**
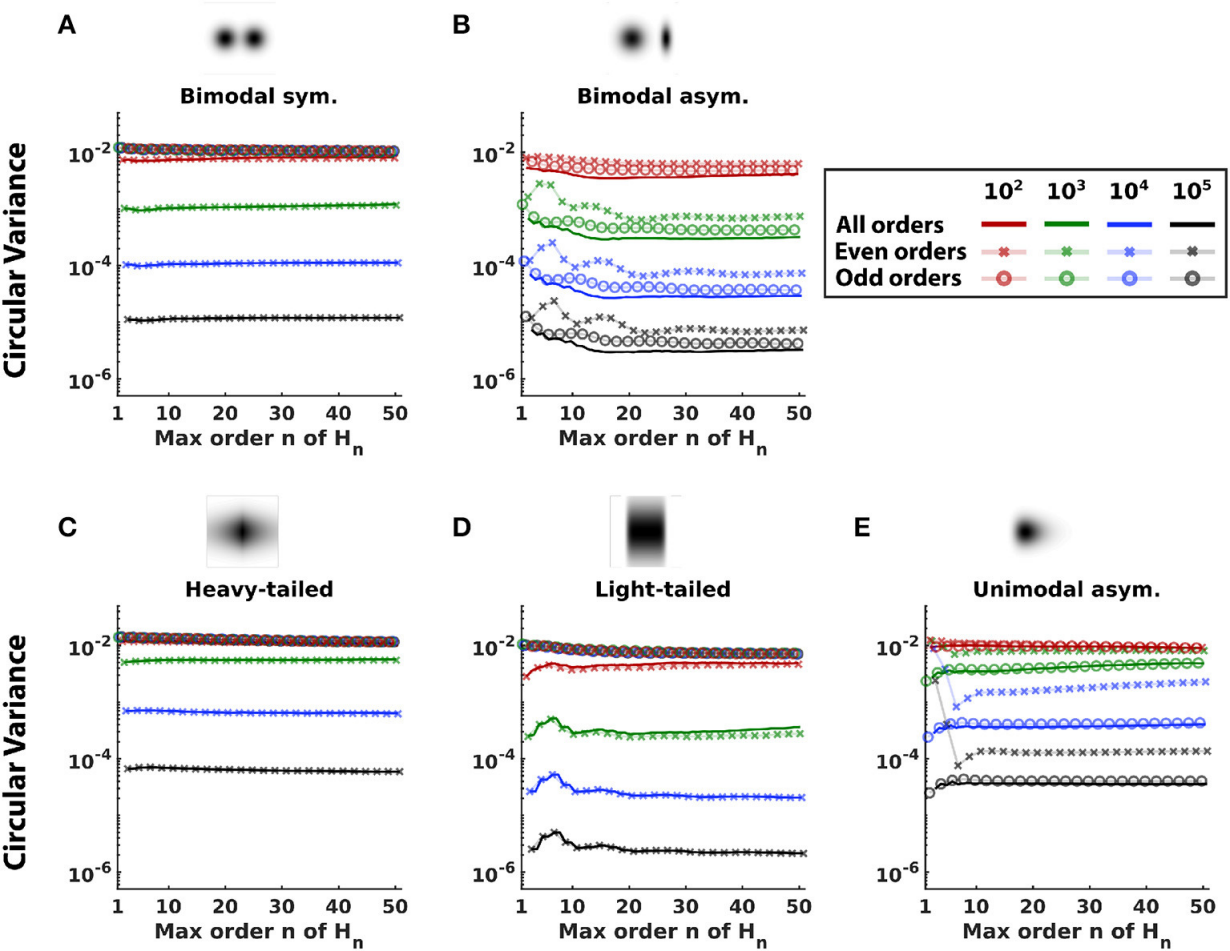
Comparison of measures of non-Gaussianity constructed with different sets of Hermite functions. Non-Gaussianity was estimated with variants of Equation (9) terminating at a range of values of *n* (solid lines), and also with variants that included only even (x connected with faint lines), or odd (o connected with faint lines) orders. Distributions are shown at the top. Colors differentiate sample sizes. Curves show the mean of circular variance, a measure of precision. **(A)** Bimodal symmetric distribution. **(B)** Bimodal asymmetric distribution. **(C)** Heavy-tailed distribution. **(D)** Light-tailed distribution. **(E)** Unimodal asymmetric distribution.

Consideration of the one-dimensional projections of 2D distributions also provides an opportunity to determine how the measure of non-Gaussianity (Equation 9) behaves as a function of *n*, since precision can be measured by exhaustive search of the projection angle. This analysis is shown in Figure 4.

For most distributions (Figures 4A–D), using all Hermite functions up to order *n* = 15 achieves minimum circular variance, or close to it. Although there are some distributions where error is minimized for a smaller *n* (unimodal asymmetric, Figure 4E) or a larger *n* (light-tailed, for large sample sizes, Figure 4D), these values of *n* have larger error for other distributions and sample sizes. On the other hand, *n* = 15 has near-optimal performance across distributions and sample sizes.

**Figure 5:**
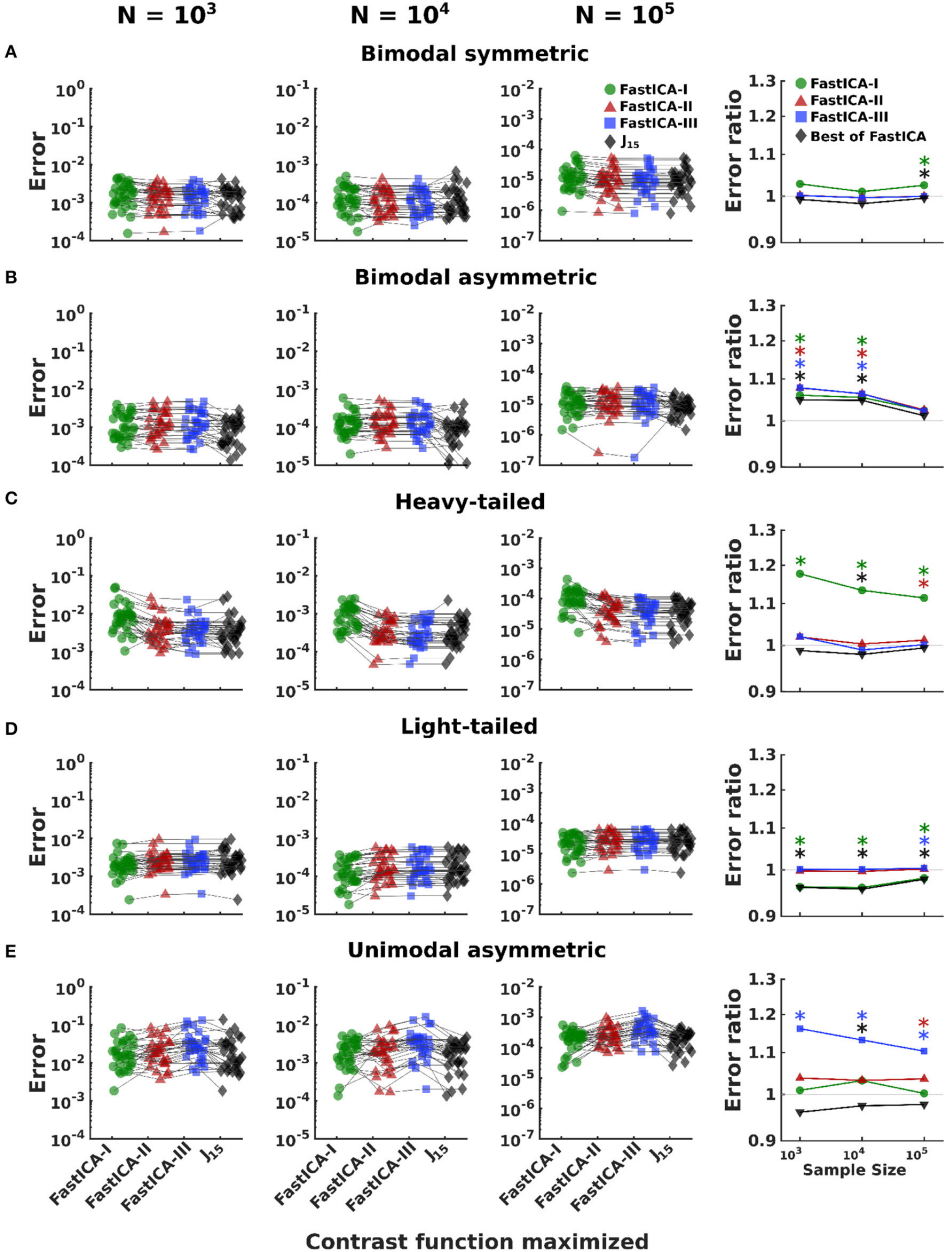
Comparison of performance of *J*_15_ to that of contrast functions of FastICA for 5D distributions. Rows **(A–E)** show results for datasets consisting of one non-Gaussian signal (the signals of Figure 3) mixed with four Gaussian signals. The first three columns show results for a range of sample sizes. For all 30 simulations of a distribution and sample size, we report the error in estimation of the direction of unmixing identified by optimizing each contrast function (see Section 2.4.1). Results for a single dataset are connected by thin black lines. The last column compares the error achieved by *J*_15_ to the three FastICA methods. Colored symbols compare *J*_15_ to each FastICA method individually. Gray symbols *J*_15_ to the best performing FastICA method for each dataset. Error ratios greater than 1 indicate that *J*_15_ has the smaller error. *Indicates significant (*p* < 0.05) differences, via a two-tailed paired *t*-test, with *p*-values computed separately for each comparison.

The analysis also shows that using all orders is preferable to variants that only use even orders or only odd orders. Performance of these variants depends on the symmetry of the distribution in a straightforward fashion. For symmetric distributions (Figures 5A, C, D), the odd-order variants perform poorly (circular variance ~1 for all *n* and all sample sizes). This is because odd-order coefficients are zero in the Hermite expansion of a symmetric distribution and do not contribute to non-Gaussianity. On the other hand, for symmetric distributions, even-order variants perform similarly to variants that use all orders (overlapping x and solid lines in Figures 5A, C, D). For asymmetric distributions (Figures 5B, E), though using all orders is better than using either only even or only odd orders, performance of the odd-order variants is better than that of the even-order variants. This is because the odd-order functions are asymmetric and thus crucial to capturing the asymmetry of the distribution. Across different sample sizes, error decreased as sample size increased but the above trends persisted.

#### 3.2.3. 5D datasets

Next, we assessed the performance of *J*_15_ for simulated 5D datasets by adding it to the FastICA package (Hyvärinen, 1999) and comparing it against the three contrast functions used for 1D and 2D datasets (Figures 2, 3). The 5D datasets were constructed by mixing a non-Gaussian distribution with four Gaussian distributions. The non-Gaussian signals were the same as in the 2D datasets (see Section 2.4). For each dataset, we implemented ICA with the three standard contrast functions and *J*_15_ and measured the error in estimating the direction of unmixing (Equation 16) when the contrast function was optimized. To optimize *J*_15_ in FastICA, we used gradient descent with a time-based learning schedule. Additionally, for all contrast functions (FastICA I-III and *J*_15_), we used 25 random starts to increase the chance of finding the global extremum.

An analysis of variance of the estimation error (Table 1) shows the expected large effects of distribution, sample size, and contrast function, and the interactions of distribution with contrast function and sample size. Sources of these effects were then explored by conducting pairwise comparisons of *J*_15_ with FastICA methods individually and to the best performing FastICA contrast function for each dataset (Figure 5).

**Table 1:** Analysis of variance of estimation error for 5D datasets.

**Source**	**Mean Sq**.	**F ratio**	***p*-value**
Distribution	544.84	818.38	< 10^−4^
Sample size	3267.34	4107.75	< 10^−4^
Contrast function	6.68	10.03	< 10^−4^
Distribution x Sample size	2.46	3.69	< 3^*^10^−4^
Distribution x Contrast function	7.19	10.80	< 10^−4^
Sample size x Contrast function	0.03	0.04	1.00
Distribution x Sample size x Contrast function	0.15	0.23	1.00

Distribution: the five shapes described in Section 2.3 and shown in Figure 3. Sample Size: three levels, 10^3^, 10^4^, and 10^5^ data points. Contrast function: the three standard FastICA contrast functions and *J*_15_. ANOVA was performed on the natural log values of the error.

Results for 5D datasets (Figure 5) follow the trends observed for 1D and 2D datasets, except that the advantages of *J*_15_ are seen only for smaller sample sizes. For bimodal symmetric distributions, *J*_15_ performs similarly to the three FastICA methods. For bimodal asymmetric distributions, *J*_15_ performs better than FastICA methods for smaller datasets (*N* = 10^3^, 10^4^) but similarly for larger sample sizes (*N* = 10^5^). For heavy-tailed distributions, the advantage of using *J*_15_ is realized for the smallest datasets (*N* = 10^3^) and it performs similarly to FastICA-II and III for larger datasets (*N* = 10^4^, 10^5^). For light-tailed distributions, FastICA-I performs best for small sample sizes and all methods perform similarly for large datasets. For unimodal asymmetric distributions, *J*_15_ performs comparably to FastICA-I and these methods outperform FastICA-II and III for all sample sizes.

#### 3.2.4. Simulated EEG datasets

An important and established use of ICA is the removal of artifact from EEG recordings (Makeig et al., 1995; Vigário, 1997; Iriarte et al., 2003). ICA is well-suited to this purpose, since many kinds of artifacts have heavy-tailed distributions. We have seen above that this is a scenario in which *J*_15_ has advantages as a test of non-Gaussianity for simple 1D distributions (Figures 1A–C), but we also saw that this advantage extended only weakly to identifying maximally non-Gaussian projections of 2D (Figure 3) and 5D distributions (Figure 5).

As these findings suggest a distinction between methods that are effective general tests of non-Gaussianity, and methods that are effective ICA contrast functions, we next tested the applicability of *J*_15_ in the use of ICA for EEG artifact removal. To do this, we generated realistic simulations of EEG datasets by using simBCI (Lindgren et al., 2018) which contained signals for 249 electrodes with ocular artifacts referred to in the paper as "eye-blinks" (Lindgren et al., 2018). Performance of *J*_15_ was assessed in the same way as for 5D datasets for sample sizes ranging from 6 s (*N* = 1.5 × 10^3^) to 5 min (*N* = 7.5 × 10^4^). An analysis of variance of the estimation error indicated large effects of sample size (*F* = 99.96, *p* < 10^−4^), contrast functions (*F* = 125.87, *p* < 10^−4^), and their interaction (*F* = 11.15, *p* < 10^−4^).

**Figure 6:**
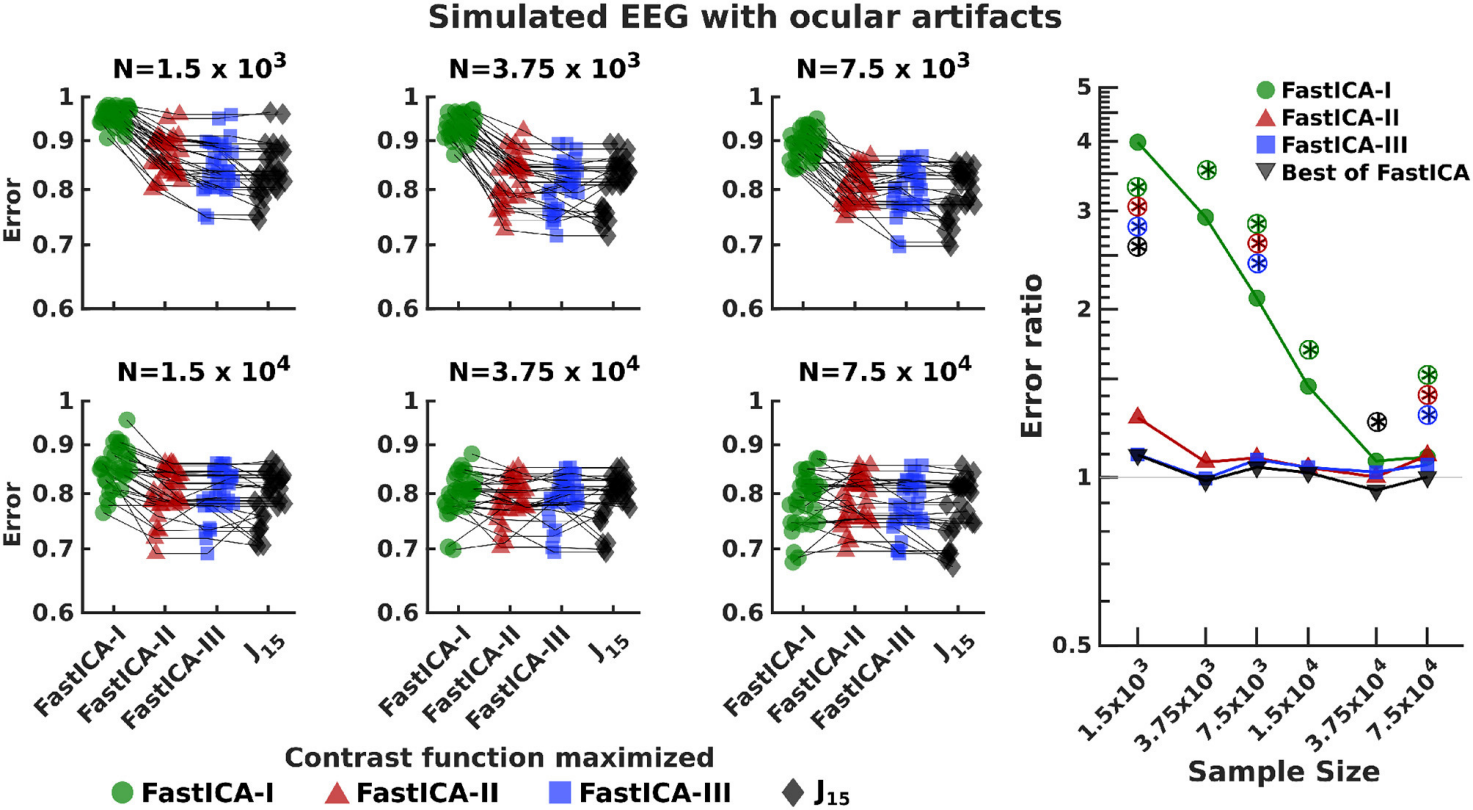
Comparison of performance of *J*_15_ to that of contrast functions of FastICA for simulated EEG datasets with ocular artifacts. The left 6 panels report the results for 30 EEG datasets generated using simBCI (Lindgren et al., 2018) at 250 Hz, analyzed for run lengths ranging from 6 s (1.5 × 10^3^ samples) to 5 min (7.5 × 10^4^ samples). Similar results were found for signals corresponding to both eyes; the average is reported. The right-most panel compares the error achieved by *J*_15_ to the three FastICA methods. Colored symbols compare *J*_15_ to each FastICA method individually. Gray symbols *J*_15_ to the best performing FastICA method for each dataset. Error ratios greater than 1 indicate that *J*_15_ has the smaller error. *Indicates significant (*p* < 0.05) differences, via a two-tailed paired *t*-test, with p-values computed separately for each comparison. Circled symbols are also significant at *p* < 0.05 false-discovery-rate correction.

Investigating the sources of these effects showed that *J*_15_ performs better than the best performing FastICA contrast function for EEG datasets of < 15 s (Figure 6). However these advantages diminish for larger datasets (*N* = 3.75 × 10^4^ and 7.5 × 10^4^, representing 2.5 and 5 min recordings respectively at 250 Hz); for these sample sizes, performance of *J*_15_ is comparable to FastICA contrast functions.

We also note that because of its implications for search strategies (see Section 2.4.1), use of *J*_15_ as a contrast function is computationally more expensive than the use of standard FastICA contrast functions. On a PC workstation (16 threads, 64 GB RAM, and 2.9 GHz clock speed), *J*_15_-based ICA of the smallest simulated EEG dataset (*N* = 1.5 × 10^3^) takes ~40 min, vs. < 30 s for FastICA contrast functions.

## 4. Discussion

We investigated the utility of a Hermite-based measure of non-Gaussianity for two purposes: as a test of normality, and as a contrast function for ICA. Compared to standard normality tests, the measure has better sensitivity to asymmetric and heavy-tailed distributions. The new method had no discernible advantage for symmetric bimodal and light-tailed distributions. To examine utility as an ICA contrast function, we assessed its ability to find the true direction of unmixing of the non-Gaussian signals. We found that its performance is largely similar to the contrast functions of a standard ICA package (FastICA), but there were modest improvements for small datasets (< 15 s) with asymmetric and heavy-tailed distributions.

The broad finding that emerged was that a method's advantages for normality tests may have only limited applicability to ICA. This discrepancy likely stems from the different goals of the two approaches: normality tests answer the question, “Is this distribution non-Gaussian at all?”, whereas the question answered by ICA is, “*Which* distribution is *the most* non-Gaussian?”. Evidence in support of this viewpoint can be found in the results for 1D bimodal distributions (Figures 1A–C). In this family, for a range of distribution shapes, the measure *J*_15_ is constant at a normalized value of 1. In other words, *J*_15_ is highly sensitive to the deviation of each of these distributions from normality, but it does not distinguish which one(s) are the most non-Gaussian. While this is not a drawback for normality tests, it is critical to ICA.

We also observed increased computational cost for *J*_15_ when used as an ICA contrast function, but not when used as a normality test. This cost arises because the fixed-point algorithm for FastICA is not applicable to *J*_15_ (see Section 2.4 for details). Thus, we used gradient descent for *J*_15_, but to counter the increased probability of getting stuck in local extrema, we augmented this strategy with a time-based learning schedule and multiple random starts. While these changes improve convergence, they increase run-time. Nevertheless, despite the increased computational cost, the improvement in accuracy achieved by *J*_15_ for small datasets make it a viable alternative.

In the context of EEG, we found that the new method has advantages over standard contrast functions for analyzing datasets spanning a few seconds. This is likely to be particularly useful in brain-computer interface applications (Xu et al., 2004; Kachenoura et al., 2007). For larger dataset (over several minutes), the method performs similarly to standard contrast functions, with a higher computational cost. We note that the current approach is specifically targeted at identifying non-Gaussian projections, and does not address statistical issues that are important in interpreting the extracted components—for example, inter-subject and inter-trial variability—which are extensively discussed in Hyvärinen (2011) and Hyvärinen and Ramkumar (2019). Moreover, since the new contrast function is used to identify one-dimensional projections in sequence, it is not immediately applicable to ICA variants that can extract multiple components simultaneously (Amari et al., 1995; Belouchrani et al., 1997).

In conclusion, the new method has advantages both as a normality test and as an ICA contrast function. As a normality test, its performance is either better than or at least comparable to commonly-used normality tests. When applied as an ICA contrast function, the new method has modest advantages for small datasets spanning over a few seconds with asymmetric and heavy-tailed distributions. In the context of EEG, this yields benefits for extraction of artifacts such as eye blinks (that have heavy-tailed distribution) in small datasets.

## Data availability statement

Code for generation of datasets, including the parameters used for generating simulated EEG datasets via the simBCI package (Lindgren et al., 2018), are available on Github at https://github.com/jvlab/hf-mong.

## Author contributions

BK contributed to conceptualization. PJ and JV contributed to methodology, formal analysis, investigation, and writing—original draft. PJ wrote the software. JV contributed to supervision and funding acquisition. All authors contributed to writing—review and editing and approved the submitted version.
